# Synthesis and Characterization of Photo-Responsive Carbosilane Dendrimers

**DOI:** 10.3390/molecules14062226

**Published:** 2009-06-18

**Authors:** Tetsuo Koyama, Ken Hatano, Koji Matsuoka, Yasuaki Esumi, Daiyo Terunuma

**Affiliations:** 1 Division of Material Science, Graduate School of Science and Engineering, Saitama University, Sakura, Saitama 338-8570, Japan; 2 The Institute of Physical and Chemical Research (RIKEN), Wako, Saitama 351-0198, Japan

**Keywords:** azobenzene, carbosilane dendrimer, heat isomerization

## Abstract

Preparation of photo-responsive carbosilane dendrimers bearing 4-phenylazo-benzonitrile units on their molecular surface has been accomplished, and their both photo and thermal behaviors have also been characterized. These functional dendrimers suggest that the apparent molecular sizes of the *cis*-isomers are smaller than those of the corresponding *trans*-isomers, since the molecular diameter of these dendrimers would be shorter on the basis of *trans*→*cis* photo-isomerization of azobenzene.

## 1. Introduction

Dendrimers are unique macromolecules with a regular, three-dimensional, spherical structure. They have attracted much attention as functional materials because they can integrate functional groups under similar conditions on their molecular surface. In our ongoing synthetic studies of carbosilane dendrimers uniformly covered with functional molecules, we have previously reported the preparation and characterization of carbosilane dendrimers carrying mesogenic groups [1] and sugar moieties [2,3,4,5,6,7].

**Figure 1:**
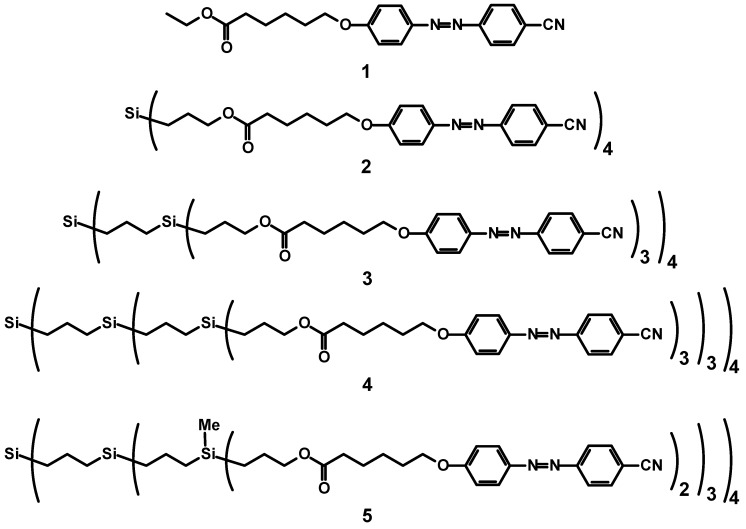
Photo-responsive compounds.

Azobenzene derivatives are well-known as photo-switchable functional moieties that undergo photo-induced (*trans*→*cis*) and thermal (*cis*→*trans*) isomerization processes [8,9]. Preparation of linear polymers bearing pendant azobenzene derivatives and a dendrimer carrying an azobenzene derivative in order to investigate the photo-responsive properties has been reported [10]. It is interesting that the molecular size of the dendrimer carrying azobenzene derivatives is controlled on the basis of photo- and heat- isomerization abilities of the azobenzene unit. Therefore, these specific molecules have potential applications in conversion of photo-energy into dynamic energy as well as in drug delivery system. In this paper, we describe the preparation and characterization of a series of new photo-responsive carbosilane dendrimers **1**-**5** uniformly functionalized with azobenzene moieties, as shown in Figure 1.

## 2. Results and Discussion

### 2.1. Synthesis of Carbosilane Dendrimer Derivatives

**Scheme 1:**
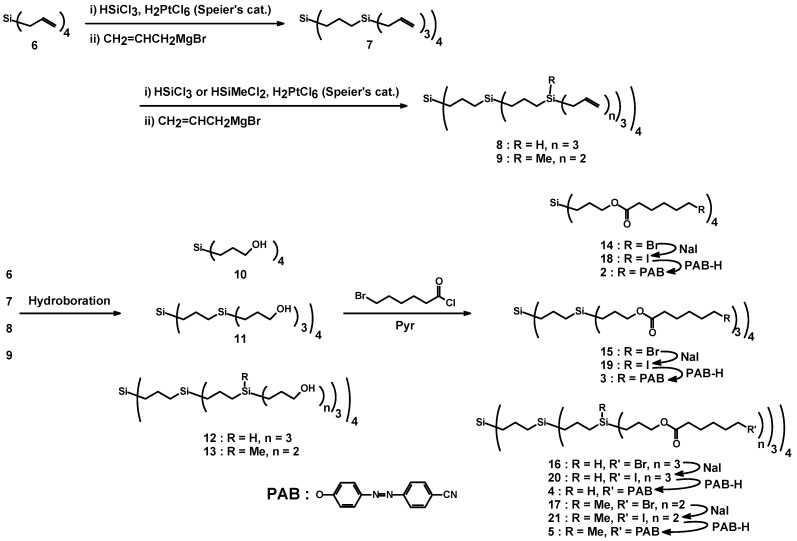
Synthetic construction of carbosilane dendrimers having photo-responsive moieties.

Scheme 1 summarizes the synthetic construction of carbosilane dendrimers having photo-responsive moieties at each terminal end. A series of carbosilane dendrimers **6-9** were prepared by the method described in the literature [11]. These dendrimers were then converted into the corresponding hydroxyl derivatives **10-13** by hydroboration reactions [12]. Then **10** was treated with 6-bromo-hexanoyl chloride in the presence of pyridine to give **14**, in which the bromine atom was next replaced by an iodine atom, giving compound **18**, by the Finkelstein reaction [13,14]. Reaction of **18** with 4-(4-hydroxy-phenylazo)benzonitrile (= PAB) was carried out in the presence of potassium carbonate to give **2** in good yield. Ethyl 6-[4(4-cyanophenylazo)phenoxy]-hexanoates **1**, **3** and **4** were synthesized in a manner similar to that described above. In contrast, the hydrosilation of **7** with HSiMeCl_2_, subsequent allylation and introduction of an azobenzene derivative gave **5**, which had a decreased number of functional groups, compared to that of **4**. 

### 2.2. Characterization of Photo-Responsive Carbosilane Dendrimers

**Figure 2:**
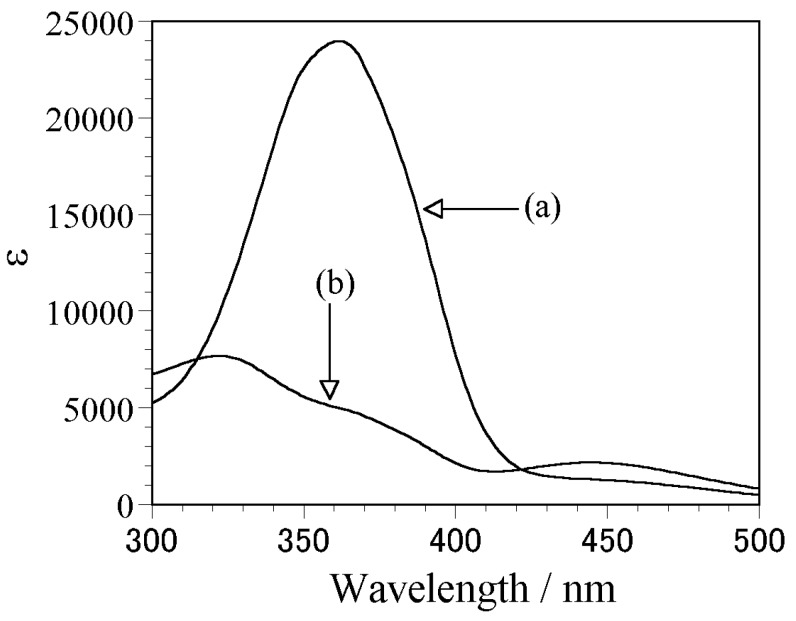
Absorption spectra of *trans*-**4** (a) and *cis*-**4** (b) in THF.

UV spectra for **4** in THF solution both before and after irradiation (360 nm) were measured at room temperature (Figure 2). An absorption maximum of the *trans*-isomer was observed at 362 nm [Figure 2(a)], and intensity of that of the *cis*-isomer decreased and the peak top of the wavelength blue-shifted to 323 nm after UV irradiation [Figure 2(b)]. However, complete transformation of the *trans*-isomer was not accomplished due to the heat irradiation from light source. The same experiments were performed for **1**, **2**, **3** and **5**, and the results of the UV absorption experiments of **1**, **2**, **3** and **5** are summarized in Table 1.

### 2.3. Analyses of the Molecular Size of the Dendrimers by GPC

It is presumed that dendrimers are globular molecules, and it was expected that the diameter of the dendrimers described in this paper would be decreased by the irradiation (360 nm) because the total length of the *cis*-azobenzene molecule is shorter than that of the *trans*-one [10]. To observe these properties, Dynamic Light Scattering (DLS) measurement is usually used. We attempted direct measurements of the dendrimers. Since the sizes of the synthesized dendrimers were smaller than the detectable limit of observation, the measurements were unfortunately not successful. We then attempted to determine the three-dimensional molecular sizes of the dendrimers by gel permeation chromatography (GPC).

*Trans*-isomers of **3** and **4** in THF solution were injected into the GPC apparatus, and the corresponding retention times were measured. The retention times of *trans* **3** and **4** were 12.87 and 11.97 min, respectively (Table 2). The weight-average molecular weights of **3** and **4** calculated from the retention times corresponded to 6,300 and 13,600, respectively, although neither of them were in agreement with the theoretical molecular weights because of the use of linear polystyrene standards for the calibration curve.

The *cis*-isomers prepared by irradiation (365 nm) were analyzed in a manner similar to that described above. The retention times of the *cis* isomers of **3** and **4** were 13.00 and 12.09 min, respectively, and the corresponding weight-average molecular weights were 5,800 and 12,300. However, it is difficult to conclude directly that these data show the difference in molecular sizes of the *trans*-isomer and the *cis*-isomer because there are complicated interactions arising from polarities between the gel and the azobenzene unit in the GPC column. It should be noted that the differences in retention times were reproducible. 

### 2.4. Thermal Isomerization Processes of Dendrimers

The thermal isomerization behavior of **1**~**5** was then investigated using ^1^H-NMR experiments. The heat-isomerization process of the *cis*-isomers was followed by placing them in a fixed temperature NMR probe (30 °C, 35 °C, 40 °C, 45 °C and 50 °C), and the *cis*→*trans* isomerization rates were calculated from the ^1^H-NMR signals in the aromatic region (*trans*-isomers: 6.7-8.2 ppm, *cis*-isomers: 6.3-7.8 ppm). The activation energies and the frequency factors were then calculated with Arrhenius plots, and the results are summarized in Table 3.

The activation energies of **2** and **3** are almost equal to that of **1,** while the activation energy of **4** is significantly higher than that of **2** and **3**. It is thought that the *cis*→*trans* thermal-isomerization is slightly prevented in the higher generation dendrimer because the azobenzene units in **4** would be in more crowded conditions and are piled up one upon another. In order to provide support for this notion, the activation energy of **5**, which has a smaller number of azobenzene units than **4**, was further measured. The *cis*→*trans* activation energy of **5** obtained was 119.7 KJ/mol, which is similar to that of **1**, **2** and **3**. 

### 2.5. Crowding of the Molecular Ends of Dendrimers

**Figure 3:**
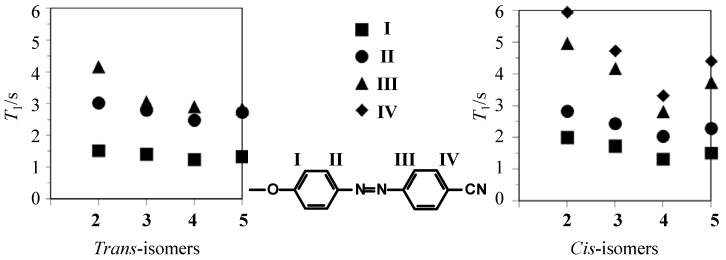
*T*_1_ values of the ^1^H on the azobenzene unit of **2**, **3**, **4** and **5** in THF-*d*_8_.

When the ends of the molecules are crowded, as suggested above, the motion of the azobenzene unit would be slowed down. Therefore, investigation of the *T*_1_ value of ^1^H on the azobenzene units was carried out (Figure 3). If *T*_1_ of **4** is smaller than that of other molecules, it would be evidence that the molecular end of **4** is crowded because a small *T*_1_ means low mobility of the unit. The highest *T*_1_ value of the ^1^H on the azobenzene unit was found for **2**, and **2**, **3** and **5** had almost equal *T*_1_ values. However, the *T*_1_ value of **4** was lower than the values of the other molecules. These results indicate that **4** has the lowest motional freedom among the dendrimers described above. 

## 3. Experimental

### 3.1. General

THF was freshly distilled from sodium just before use. For UV irradiation, a 400 W High-Pressure Mercury Light with a glass filter (Toshiba UV-36C) was used as the light source. ^1^H-NMR and ^13^C-NMR were recorded on a Bruker ARX-400 spectrometer operating at 400 and 100 MHz, respectively. Abbreviations of G1 as first generation structure of dendrimers and G2 as 2^nd^ ones were used in NMR assignments. The preparative GPC system was used by recycle-type GPC with a tandem gel-column (JAIGEL 3H+2.5H: 20φ × 600 mm), using a differential refractive index detector (RI-50). The analytical GPC was a Shimadzu LC-VP with a polystyrene standard (ShimPack GCP 0825+802; flow rate, 1.0 mL/min; oven temp., 30 °C). UV-spectrum measurement was performed with a JASCO V550 U/V-spectrometer. Matrix-assisted laser desorption/ionization time-of-flight mass spectra (MALDI-TOF-MS) were obtained using a Perseptive Biosystems Voyager Elite spectrometer. Melting point measurement was performed with a Büchi B-540 Melting Point apparatus.

*Ethyl 6-[4-(4-cyanophenylazo)phenoxy]hexanoate* (**1**). A solution of ethyl 6-bromohexanoate (1.98 g, 8.87 mmol) in 2-butanone (EMK) (30 mL) was added to a mixture of 4-(4-hydroxyphenylazo)-benzonitrile (1.78 g, 7.96 mmol) and K_2_CO_3_ (1.23 g, 8.88 mol) in EMK (30 mL). The mixture was stirred at 90 °C for 24 h. After the precipitates had been filtered off, THF (50 mL) was added to the filtrate and the solution was washed with water (50 mL × 3). The extract was dried over anhydrous Na_2_SO_4_. The solvent was evaporated, and the resulting red crystals were recrystallized from EMK to give 2.48 g (85.4%) of pure **1**: mp 133.0-133.6 °C; ^1^H-NMR (CDCl_3_) δ 1.2-1.3 (t, 3 H, CH_3_), 1.5-1.8 (m, 6 H, CH_2_), 2.3-2.4 (t, 2 H, C(O)CH_2_), 4.0-4.1 (t, 2 H, CH_2_-O), 4.1-4.2 (q, 2 H, CH_3_C*H_2_*), 7.0-8.0 (m, 8 H, Ar); ^13^C-NMR (CDCl_3_) δ 14.2 (CH_3_), 24.6 (OCOCH_2_*C*H_2_), 25.6 (*C*H_2_CH_2_CH_2_-O), 28.7 (CH_2_*C*H_2_CH_2_-O), 34.2 (OCO*C*H_2_), 60.3 (CH_3_*C*H_2_), 68.0 (*C*H_2_-O-PAB), 114-133 (Ar).

*Tetrakis[3-{6-[4-(4-cyanophenylazo)phenoxy]hexanoyloxy}propyl]silane* (**2**). A solution of tetrakis[3-(6-iodohexanoyloxy)propyl]silane **18** (141 mg 0.115 mmol) in EMK (30 mL) was added to a mixture of 4-(4-hydroxyphenylazo)benzonitrile (154 mg 0.690 mmol) and K_2_CO_3_ (105 mg 0.761 mmol). The mixture was stirred at 90 °C for 48 h. THF (50 mL) was added to the mixture, and the suspension was washed with water (50 mL × 3). The organic solution was dried over anhydrous Na_2_SO_4_. The solvent was evaporated and purified by preparative GPC with THF as the eluent to give 135 mg (73.4%) of **2**: ^1^H-NMR (CDCl_3_) δ 0.45-0.65 (br, 8 H, SiCH_2_), 1.20-1.80 (m, 32 H, CH_2_ (G0, G1)), 2.30-2.45 (t, 8 H, COCH_2_), 4.00-4.15 (d, 16 H, CH_2_-O), 6.95-8.05(m, 32 H, Ar); ^13^C-NMR (CDCl_3_) δ 173-114 (Ar), 68.0 (CH_2_), 66.7 (CH_2_), 34.1 (CH_2_), 28.8 (CH_2_), 25.6 (CH_2_), 24.6 (CH_2_), 23.0 (CH_2_), 7.86 (SiCH_2_).

*Tetrakis{3-[tris(3-{6-[4-(4-cyanophenylazo)phenoxy]hexanoyloxy}propyl)silyl]propyl}silane* (**3**). Compound **3** was prepared in a manner similar to that described for **2**: ^1^H-NMR (CDCl_3_) δ 0.50-0.70 (m, 40 H, SiCH_2_ (G0, G1)), 1.20-1.40 (m, 8 H, CH_2_ (G0)), 1.40-1.90 (m, 96 H, CH_2_ (G1)), 2.30-2.40 (t, 24 H, COCH_2_), 3.90-4.10 (m, 48 H, CH_2_-O), 6.90-7.80 (m, 96 H, Ar); ^13^C-NMR (CDCl_3_) δ 173-114 (Ar), 118 (C=O), 113 (CN), 68.0 (CH_2_, G1), 66.7 (CH_2_, G1), 34.0 (CH_2_, G1), 28.9 (CH_2_, G1), 25.6 (CH_2_, G1), 24.7 (CH_2_, G1), 23.2 (CH_2_, G1), 18.4 (CH_2_, G0), 7.99 (SiCH_2_, G0, G1).

*Tetrakis[3-(tris{3-[tris(3-{6-[4-(4-cyanophynylazo)phenoxy]hexanoyloxy}propyl)silyl]propyl}silyl)-propyl]silane* (**4**). Compound **4** was prepared in a manner similar to that described for **2**: ^1^H-NMR (CDCl_3_) δ 0.45-0.78 (br, 136 H, SiCH_2_ (G0, G1, G2)), 1.20-1.80 (m, 320 H, CH_2_), 2.20-2.40 (t, 72 H, COCH_2_), 3.85-4.10 (d, 144 H, CH_2_-O), 6.80-7.90 (m, 288 H, Ar); ^13^C-NMR (CDCl_3_) δ 173-114 (Ar), 128 (C=O), 113 (CN), 68.0 (CH_2_, G2), 66.7 (CH_2_, G2), 34.0 (CH_2_, G2), 28.9 (CH_2_, G2), 25.6 (CH_2_, G2), 24.7 (CH_2_, G2), 23.2 (CH_2_, G2), 18.8 (CH_2_, G0, G1), 7.99 (SiCH_2_, G0, G1, G2); MALDI-TOF mass Found: *m/z* 14797.0. Calcd. for C_840_H_960_N_108_O_108_Si_17_Na: [M+Na]^+^, 14797.7.

*Tetrakis[3-(tris{3-[bis(3-{6-[4-(4-cyanophynylazo)phenoxy]hexanoyloxy}propyl)methylsilyl]propyl}-silyl)propyl]silane* (**5**). Compound **5** was prepared in a manner similar to that described for **2**. ^1^H-NMR (CDCl_3_) δ -0.10-0.10 (s, 36 H, SiCH_3_), 0.40-0.80 (br m, 112 H, SiCH_2_ (G0, G1, G2)), 1.20-2.00 (m, 224 H, CH_2_), 2.20-2.40 (t, 48 H, COCH_2_), 3.85-4.10 (d, 96 H, CH_2_-O), 6.80-8.00 (m, 192 H, Ar); ^13^C-NMR (CDCl_3_) δ 173-114 (Ar), 128 (C=O), 113 (CN), 68.0 (CH_2_, G2), 66.7 (CH_2_, G2), 34.0 (CH_2_, G2), 28.9 (CH_2_, G2), 25.6 (CH_2_, G2), 24.7 (CH_2_, G2), 23.2 (CH_2_, G2), 18.8 (CH_2_, G0, G1), 9.50 (SiCH_2_, G0, G1, G2), -5.30 (SiCH_3_); MALDI-TOF mass Found: *m/z* 10436.6. Calcd. for C_588_H_708_N_72_O_72_Si_17_Na: [M+Na]^+^, 10436.8.

## 4. Conclusions

Although various azobenzene-substituted dendrimers have been prepared in terms of their photo-responsiveness [15,16,17,18,19], there have been few studies on the problems generated from their conformation. In this paper, we have described the preparation of a series of carbosilane dendrimers having photo-sensitive functionalities and their isomerization process. The results of this study suggested that the densities of the molecules influence their isomerization.

## Figures and Tables

**Table 1:** Results of UV absorption of **1**, **2**, **3**, **4** and **5**.

Compounds	*Trans*-isomer		*cis*-isomer	
	λ_max_(nm)	ε^a)^(M^-1^ cm^-1^)	λ_max_(nm)	ε ^a)^(M^-1^ cm^-1^)
**1**	364	2.99×10^4^	327	1.06×10^4^
**2**	364	2.56×10^4^	320	7.06×10^3^
**3**	362	2.40×10^4^	323	7.67×10^3^
**4**	366	2.92×10^4^	324	9.61×10^3^
**5**	363	2.26×10^4^	321	8.55×10^3^

^a^The concentrations and ε were estimated from the value on the basis of a 4-(phenylazo)benzonitrile molecule in THF solution.

**Table 2:** Results of GPC analyses^a^^)^ of **3** and **4**.

Compounds	Retention time (min)	
	*Trans*	*Cis*
**3**	12.87	13.00
**4**	11.97	12.09

^a)^ Analytical conditions; Flow rate: 1.0 mL/min; Oven temp.: 30 °C.

**Table 3:** Results of kinetic experiments^a)^ for the isomerization.

Compounds	Activation energy	Frequency factor
	(KJ / mol)	ln(*A* / s^-1^)
**1**	119.3	37.66
**2**	120.5	37.95
**3**	120.1	37.74
**4**	132.3	42.75
**5**	119.7	38.04

^a^THF-*d*_8_ solutions of **1**, **2**, **3**, **4** and **5** adjusting the concentration of the azobenzene unit to 1.0×10^-2^ M.
